# Nonlinear dynamics of large-scale activity in "networks of networks"

**DOI:** 10.1186/1471-2202-14-S1-P331

**Published:** 2013-07-08

**Authors:** Fereshteh Lagzi, Fatihcan M Atay, Stefan Rotter

**Affiliations:** 1 Bernstein Center Freiburg & Faculty of Biology, University of Freiburg, Germany; 2 Max Planck Institute for Mathematics in the Sciences Leipzig, Germany

## 

As a first step toward understanding the macro-dynamics of brain-like systems, we study the large-scale dynamics of balanced random networks of excitatory and inhibitory integrate-and-fire neurons. Based on the dynamical equations of the model, a mean field approach was previously employed to reduce the dimensionality of the network dynamics [1,2]. Here, we analyze the joint activity dynamics of excitatory and inhibitory populations employing a pair of mutually interacting nonlinear differential equations. In absence of a voltage leak for individual neurons, and for negligible synaptic transmission delay, these equations take the form of Lotka-Volterra equations. These have been used to describe predator-prey systems, corresponding to excitatory and inhibitory populations of neurons in our case. For non-zero identical synaptic transmission delay, we obtain Lotka-Volterra equations with delay. We try to infer the parameters for the non-autonomous differential equations given a dataset from numerical simulations of such a network. Moreover, we attempt to analytically constrain the parameters and compare them with their statistical estimators. Using simulation results, the significance of the nonlinear dynamics becomes obvious in the vector field of excitatory-inhibitory activity, which corresponds nicely with the vector field of the analytical equations.

We have analyzed the stability of the network considering two bifurcation parameters: the relative strength of recurrent inhibition, "g", which controls the balance between excitation and inhibition in the network, and the intensity of external input to the network, "η". We have found out that for a value of "g" that keeps the exact balance between excitation and inhibition, a bifurcation from unstable to stable network dynamics takes place. This bifurcation separates Synchronous Regular (SR) from Asynchronous Irregular (AI) activity of the network, similar to what was found in a previous study on the same network using a Fokker-Planck approach [3]. The influence of synaptic delays on the reduced dynamics of the network is currently under study.

It has been shown that Lotka-Volterra equations are capable of representing switching dynamics between different states of neural networks [2,4]. Our analysis represents a first step toward analyzing the dynamics of more complex "networks of networks" that are implicated in various cognitive abilities of the brain.
